# Hybrid System Coupling Moving Bed Bioreactor with UV/O_3_ Oxidation and Membrane Separation Units for Treatment of Industrial Laundry Wastewater

**DOI:** 10.3390/ma13112648

**Published:** 2020-06-10

**Authors:** Sylwia Mozia, Magdalena Janus, Sławomira Bering, Krzysztof Tarnowski, Jacek Mazur, Kacper Szymański, Antoni W. Morawski

**Affiliations:** 1 Faculty of Chemical Technology and Engineering, West Pomeranian University of Technology in Szczecin, Pułaskiego 10, 70-322 Szczecin, Poland; kacper.szymanski@zut.edu.pl (K.S.); antoni.morawski@zut.edu.pl (A.W.M.); 2 Faculty of Civil Engineering and Architecture, West Pomeranian University of Technology in Szczecin, Piastów 50, 70-311 Szczecin, Poland; slawomira.bering@zut.edu.pl (S.B.); krzysztof.tarnowski@zut.edu.pl (K.T.); jacek.mazur@zut.edu.pl (J.M.)

**Keywords:** laundry wastewater, microfiltration, nanofiltration, advanced oxidation process, ozonation

## Abstract

This paper describes the investigations on the possibilities of treatment of wastewater generated in an industrial laundry with application of a combined biological-photooxidation- membrane system aimed at water recycle and reuse. The two treatment schemes were compared: 1) scheme A consisting of a treatment in a moving bed biological reactor (MBBR) followed by microfiltration (MF) and nanofiltration (NF), and 2) scheme B comprising MBBR followed by oxidation by photolysis enhanced with in situ generated O_3_ (UV/O_3_) after which MF and NF were applied. The removal efficiency in MBBR reached 95–97% for the biochemical oxygen demand; 90–93% for the chemical oxygen demand and 89–99% for an anionic and a nonionic surfactants. The application of UV/O_3_ system allowed to decrease the content of the total organic carbon by 68% after 36 h of operation with a mineralization rate of 0.36 mg/L·h. Due to UV/O_3_ pretreatment, a significant mitigation of membrane fouling in the case of both MF and NF processes was achieved. The MF permeate flux in the system B was over two times higher compared to that in the system A. Based on the obtained results it was concluded that the laundry wastewater pretreated in the MBBR-UV/O_3_-MF-NF system could be recycled to any stage of the laundry process.

## 1. Introduction

One of the significant operating costs of an industrial laundry is water and wastewater management. Because of that, laundry investment plans should take into account an improvement of wastewater treatment and water reuse systems [1,2]. Therefore, research in this field is necessary for the optimal design of water and wastewater management facilities.

Laundry wastewater exhibits a very complex composition. It can contain fats, dyes, suspended solids, salts, organic matter, surfactants, solvents, plasticizers, emulsifiers, and even pathogens [3]. The quality of laundry wastewater depends on the washing assortment, the amount of water used, and the washing agents. The specific pollutants present in the laundry wastewater are surfactants [3,4]. Hence, wastewater from laundries needs the application of complicated treatment systems, most commonly multistep ones. The conventional physicochemical methods of treatment of laundry wastewater, such as filtration-coagulation-sedimentation, generally exhibit low effectiveness [5]. One alternative can be application of membrane technology, especially pressure driven techniques such as microfiltration (MF) or ultrafiltration (UF). A system combining coagulation followed by MF was found to be efficient in removal of total suspended solids (TSS, 93%), chemical oxygen demand (COD, 74%) and total phosphorus (22%) [6]. A complete removal of turbidity and 55–65% decrease of COD value was also reported in other studies on the combined coagulation-MF process [7]. The treatment of laundry wastewater with UF alone was found to be efficient with reference to turbidity and COD removal (>90% and >80%, respectively), but the permeate still contained both organic and inorganic dissolved contaminants [8].

To improve the quality of the product (MF/UF permeate) the application of more advanced treatment schemes is necessary. One proposed approach is a method based on preliminary coagulation, followed by dissolved air flotation (DAF), sand filtration, ozonation, granulated activated carbon (GAC) filtration, and finally ultrafiltration. It was reported that the total content of surfactants in the outflow wastewater treated in such a system did not exceed 2 mg/L [9]. According to the authors, the obtained UF permeate could be possibly used in some washing processes of home textiles. A sequential process [10] of coagulation/flocculation/sedimentation-adsorption-MF applied for treatment of the effluent from an industrial laundry was able to obtain a removal efficiency of 99.9% for color, 80% for COD, 92.9% for surfactants, and 99.4% for turbidity [10]. To evaluate the possibility of reuse of laundry wastewater, a polishing of UF permeate by either adsorption, or nanofiltration (NF), or electrooxidation was investigated [11]. It was found that surfactants present in UF permeate were effectively removed by adsorption on GAC or NF. The product quality with reference to TSS, turbidity, COD, and surfactants met environmental requirements and reusability standards.

Another attempt was based on the utilization of biological technologies such as membrane bioreactors (MBR) [1,12,13,14], sequencing batch reactors (SBR) [4], or moving bed bioreactors (MBBR) [15,16,17]. MBR and MBBR technologies were found to be effective in the treatment of wastewater containing detergents both when applied as a stand-alone process or as the first stage before laundry water renewal processes [1,18,19,20]. It was found that the laundry wastewater treated in MBBR met the quality standards required by law for wastewater discharged to surface waters [16,17]. Furthermore, it was reported that the MBR effluent could be directly reused as a process water, although only when low or medium water quality is required [1]. To improve the product quality final polishing of MBR effluent by reverse osmosis (RO) was proposed in a commercial laundry in Darmstadt, Germany [1,19]. A membrane bioreactor (MBR) characterized by high nitrogen removal efficiencies was also proposed for treatment of another type of real wastewater-landfill leachate of various concentrations (50%, 75%, and 100% v/v) corresponding to different organic loading rates. Proteinous and carbohydrate extracellular polymeric substances (EPS) and soluble microbial product (SMP) were strongly correlated (p < 0.01) with organic load, salinity and NH_4_^+^-N [21].

Although membrane processes, especially pressure driven membrane techniques, are considered as one of the best solutions for the reuse of water [18,22], they exhibit some drawbacks. The membranes applied in MF or UF suffer from fouling. This undesired phenomenon is associated with deposition of contaminants present in feed on a membrane surface or within its pores. That leads to a decrease of permeate flux and, as a result, to an increase of operational costs related to membrane cleaning and energy usage. One solution for the fouling problem could be application of advanced oxidation processes (AOPs), such as photo-Fenton, H_2_O_2_ oxidation, TiO_2_ photocatalysis, or combined UV/O_3_ and UV/TiO_2_/O_3_ systems [18,23]. Several researchers confirmed a positive influence of applying various AOPs on membrane fouling mitigation [17,23,24,25,26,27,28,29,30,31].

Based on the results presented in [3,18] the coupling of biological methods with membrane separation and AOPs was found as a promising solution for water renewal in an industrial laundry. Two hybrid systems utilizing (i) MBBR, UV/O_3_, and MF followed by NF [3] and (ii) hybrid MBBR (HMBBR), UV/O_3_, and UF followed by NF [18], were investigated. The time of AOP pretreatment, as an important factor influencing MF and UF permeate flux in terms of fouling mitigation, was reported [3,18]. It was also found that the time of UV/O_3_ pretreatment had no significant influence on the quality of NF permeate [3,18].

In the present paper, two multistage systems for treatment and reuse of laundry wastewater are compared. In the first step, the wastewater was treated in a moving bed bioreactor. Thereafter, the pretreated wastewater was supplied to one of the following systems: (a) system A: MF followed by NF or (b) system B: photolysis enhanced with in situ generated O_3_ (UV/O_3_) followed by MF and NF. The influence of the wastewater pretreatment procedure on MF and NF permeate flux and quality at various volume concentration ratio (VCR) values was investigated. The possibilities of NF permeate recycling to the laundry process were also evaluated.

## 2. Materials and Methods

The wastewater was obtained from the industrial laundry Albatros Sp. z o. o. Sp. k. (Nowe Czarnowo, Poland). The laundry washes about 80 tons of linens and generates on average 600 m^3^/d of industrial wastewater. The laundry wastewater mainly contains surfactants used for wet washing and impurities originating from washed fabrics. The wastewater from the washing process is mixed with that from the regeneration of ion exchangers, which contains high concentrations of Na^+^ and Cl^−^ ions. The surfactants used in the laundry meet the biodegradation criteria described in the regulation (EC) no. 648/2004 of the European Parliament and the Council of 31 March 2004 on detergents.

**Figure 1:**
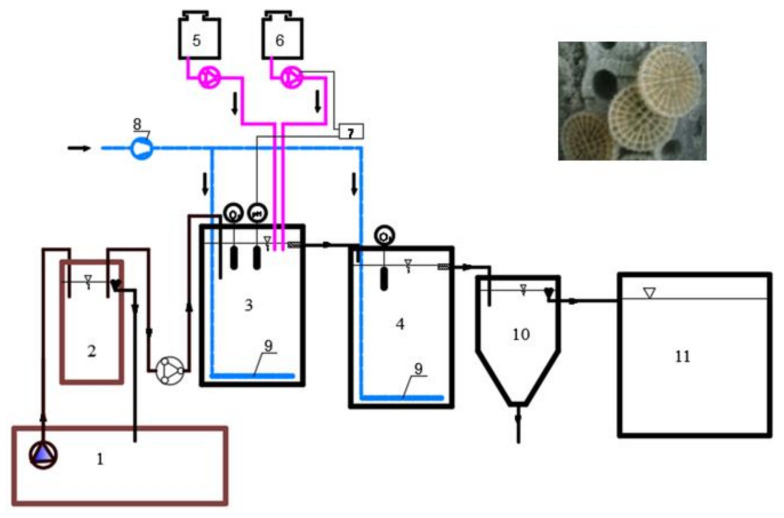
Scheme of MBBR and a photograph of Kaldnes K5 carriers. Legend: 1—equalization tank (350 m^3^); 2—buffer tank; 3—MBBR unit-1st stage; 4—MBBR unit-2nd stage; 5—urea dispenser; 6—acid dispenser; 7—pH sensor; 8—blower; 9—diffuser; 10—secondary settling tank; 11—treated wastewater tank.

The biological treatment was performed in a two-stage pilot scale plant (Figure 1) localized on place in the industrial laundry and continuously fed with real averaged wastewater pumped from a 350 m^3^ volume retention tank. The total capacity of the two MBBR units was equal to 400 L. Both MBBR reactors worked under aerobic conditions with a coarse bubble aeration system. Kaldnes K5 carriers (specific area, 800 m^2^/m^3^) were applied as a biofilm support. The carriers floating in the wastewater were overgrown with microorganisms, typical for an active sludge and a biological bed.

The MBBR daily flow Q_d_ was equal to 0.6–0.8 m^3^/d (mean hourly flow Q_h_ = 25–33 L/h). Hydraulic retention time (HRT) was in the range of 12–16 h. The concentration of dissolved oxygen in the MBBR tanks was maintained on the level of 2–4 mgO_2_/L. To improve the conditions of biological treatment, the aqueous solution of urea (commercial Ad-blue™ (Grupa Azoty, Police, Poland) solution containing 165.3 g/L of nitrogen diluted with water) was dosed to the raw wastewater. The required loading of nitrogen was equal to 5 mgN/L. Based on pH measurements in the first MBBR tank, an aqueous solution of H_2_SO_4_ (ca. 10%) was used for automated pH control (when above pH 8). The operating profiles of the MBBR were discussed in details in the previous studies [17]. The MBBR effluent applied in the next steps of the experiments was collected in an equalization tank until a desired volume was obtained.

The MBBR effluent was supplied to MF followed by NF system with or without pretreatment in a UV/O_3_ photoreactor. The schemes of the membrane installation and the photoreactor can be found elsewhere [3,18]. In case of UV/O_3_ pretreatment (system B) the effluent from MBBR was supplied to a wastewater tank (1.8 m^3^) from which it was pumped to a photoreactor equipped with a UV–vis mercury lamp (Ultralight AG, Schaanwald, Liechtenstein, 6 kW, UV intensity: ca. 330 W/m^2^). Under the action of the UV–vis irradiation a small amount of O_3_ was generated in situ, which enhanced the mineralization of organic contaminants. The concentration of ozone in water amounted to ~30 μg/L. The process was carried out in a batch mode with a complete recycling to the wastewater tank. During the UV/O_3_ treatment the wastewater was continuously aerated. After 36 h of UV/O_3_ process (time selected on a basis of the previous research [18]), the pretreated effluent was collected from the installation and further applied as MF feed. In the experiment without UV/O_3_ pretreatment (system A), the MBBR effluent was directly used as MF feed.

During MF a ceramic Eternium^TM^ membrane (pore size: 0.14 μm; filtration area 0.35 m^2^) was applied. In the first stage of the investigations, the influence of transmembrane pressure (TMP) on MF permeate flux was investigated. Thus, both MF retentate and permeate were recycled to the feed tank. In the second stage of the research the effect of volume concentration ratio (VCR) on MF permeate flux and quality was evaluated. During these experiments, the MF retentate was recycled to the feed tank, while the defined volume of permeate was collected in the permeate tank to reach the VCR values of 2–10. Both stages of the investigations were realized in systems A and B. The MF experiments were conducted at TMP = 0.5–3 bar and feed cross flow velocity of 4.5 m/s. The feed temperature was kept at 20 ± 1 °C.

**Figure 2:**
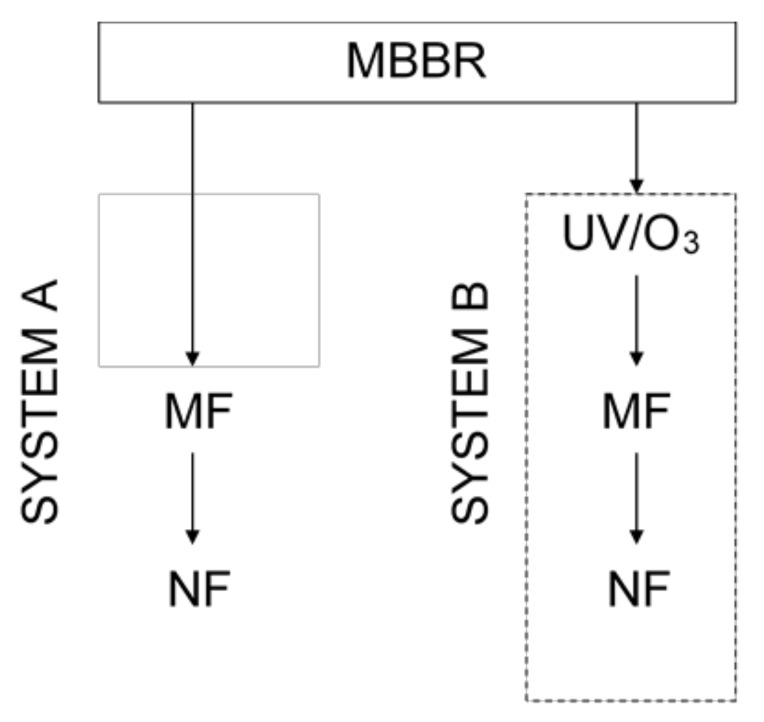
A scheme of the procedure of the treatment of laundry wastewater in systems A and B.

The MF permeate was further applied as NF feed. A commercial DOW FILMTEC NF90-2540 polymeric membrane (DuPont Water Solutions, Delfgauw, Netherlands) (2.6 m^2^) was used. NF was run at TMP = 15 bar and feed flow rate of 1.2 m^3^/h. The feed temperature was kept at 20 ± 1 °C. Similarly, as in case of MF, the influence of VCR in the range of 2–10, on the permeate flux and quality was investigated. The treatment scheme is summarized in Figure 2.

The concentration of total phosphorus and nitrogen, anionic and non-ionic surfactants, as well as the COD and biochemical oxygen demand (BOD_5_) values, were determined using Hach Lange cuvette test and the DR2800 spectrophotometer (Hach Company, Loveland, CO, USA). The concentration of the total organic carbon (TOC), total inorganic carbon (TIC), and total carbon (TC) was measured using IL550 TOC-TN analyzer (Hach Lange, Loveland, CO, USA). The inorganic ions content was determined on a basis of ion chromatography (850 Professional IC, Metrohm, Herisau, Switzerland). A Metrohm A Supp5-250 analytical column in series with a Metrosep RP guard column and a carbonate-based eluent (Na_2_CO_3_ + NaHCO_3_) were applied for anions measurement, while a Metrosep C2-150 analytical column in series with a C2 guard column and a mixture of tartaric acid with 2-picoline acid were used for cation analysis. The conductivity and the total dissolved solids (TDS) content were measured using Ultrameter™ 6P (Myron L Company, Carlsbad, CA, USA). The pH was determined with application of the CP-105 pH meter (Elmetron, Zabrze, Poland). The turbidity was measured with 2100N IS turbidimeter (Hach Lange, Loveland, CO, USA). All measurements were repeated two times and the presented results are mean values from these data.

## 3. Results and Discussion

The first stage of the treatment of the laundry wastewater was realized in the MBBR pilot plant. Table 1 summarizes the composition of the wastewater before and after the treatment. The application of the biological system resulted in a high efficiency of the removal of organic contaminants measured as BOD_5_ (95–97%) and COD (90–93%). Moreover, the concentration of the anionic and nonionic surfactants was lowered by 89–99%, showing a good performance of the applied MBBR towards these substances. The treatment of the laundry wastewater in MBBR utilizing Kaldnes K5 carriers was already discussed in detail in the previous work [16], in which the results of the long-term operation (over 4 months) of MBBR unit were presented. The data summarized in Table 1 confirm the overall high treatment efficiency of the laundry wastewater in the biological process applied. A similar efficiency (95%) of the removal of a nonionic surfactant, alcohol ethoxylate, in a submerged anaerobic membrane reactor (SAnMBR) was reported by Chen et al. [32], while the removal of an anionic surfactant, linear alkylbenzene sulfonate (LAS), was significantly lower (44%). Similarly, low effectiveness of LAS removal (~50%) from the laundry wastewater in an up-flow anaerobic sludge blanket (UASB) reactor was recently reported by Delforno et al. [33]. A more efficient removal (80%) of an anionic surfactant from the textile wastewater was described in the work by Bulc and Ojstrsek [34] on a treatment plant based on wetlands with *Phragmites australis*. The above literature data confirm a superior performance of the proposed MBBR system for the treatment of the industrial laundry wastewater.

Nonetheless, despite the high removal of surfactants, the MBBR effluent still contained some amounts of these contaminants. In the case of the effluent applied in the present research, the concentrations of the non-ionic and anionic surfactants were in the range of 0.1–2.4 mg/L (Table 1). Since these substances are regarded as the contaminants of emerging concern (CECs) [35] they should not be present in wastewater entering the environment even at very low loadings.

Moreover, a direct reuse of the MBBR effluent in the laundry process was not possible because it still contained too high amount of the organic contaminants (~19 mg TOC/L) and exhibited high conductivity (1800–2000 μS/cm) as well as turbidity (~7 NTU). Table 2 presents the composition of MBBR effluent applied in systems A and B. In system A the effluent was post-treated using MF followed by NF. When the wastewater was treated in system B, the UV/O_3_ process was used before MF and NF filtration. The composition of the wastewater applied during UV/O_3_, MF, and NF was monitored by measuring TOC, TIC, conductivity, and concentration of the following ions: Cl^−^, SO_4_^2−^, Na^+^, K^+^ Ca^2+^, and Mg^2+^.

**Figure 3:**
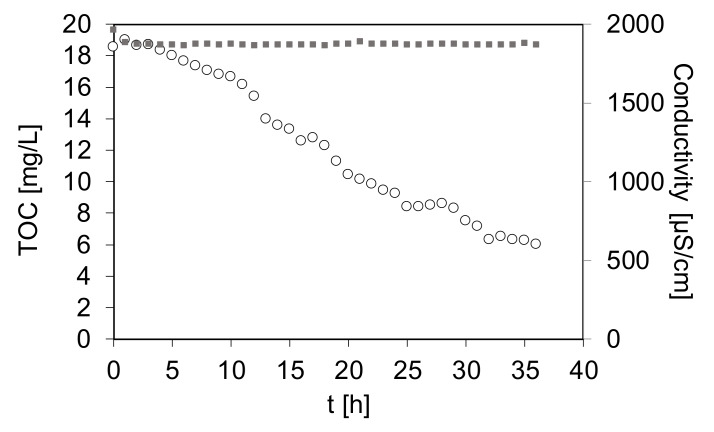
Mineralization of organic contaminants measured as TOC and changes of effluent conductivity during UV/O_3_ process with in situ formed O_3_ applied to the wastewater pretreated in MBBR.

MF was proposed as a process of the turbidity removal, while NF as a final polishing step allowing for rejection of low molecular organic contaminants as well as inorganic salts (especially Ca^2+^ and Mg^2+^ compounds responsible for hardness, as well as NaCl from regeneration of ion exchangers). Nonetheless, since it is known that MF membranes are prone to fouling, in system B the additional treatment of the MBBR effluent by UV/O_3_ oxidation with in situ formed O_3_ was employed in order to decrease the content of organic matter. In the previous investigations [18] it was found that UV/O_3_ treatment realized for at least 36 h allowed to considerably reduce membrane fouling during ultrafiltration and conduct the process without any significant deterioration of the membrane permeability. During 36 h of the photodegradation process (Figure 3) the mineralization of the organic contaminants proceeded continuously and at the end of the treatment the concentration of TOC was equal to 6 mg/L (68% removal).

The mineralization rate calculated for this process amounted to 0.36 mg/L·h. The data presented in Figure 3 show that conductivity was constant during the whole experiment, indicating that the mineralization of such low TOC content did not affect the value of this parameter. Furthermore, a decrease of the turbidity down to 2 NTU and a slight increase of pH (from 8.4 to 9.0) was observed.

**Figure 4:**
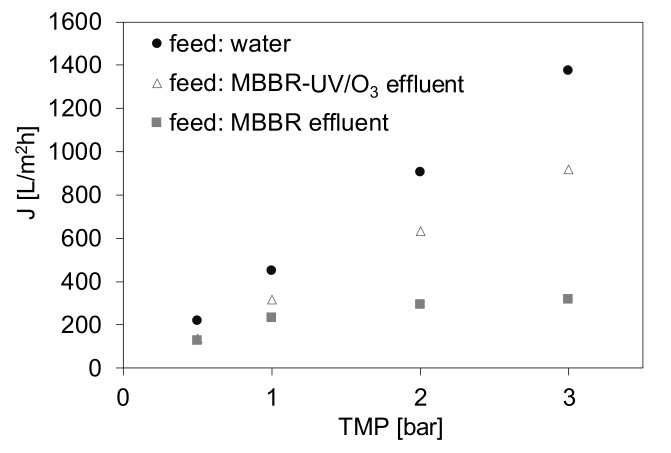
Influence of UV/O_3_ treatment on MF permeate flux.

In the next stage of the research the as-received MBBR effluent and the effluent pretreated in the UV/O_3_ process were used as MF feed. The permeate fluxes measured at various TMP during filtration of the effluents in comparison to pure water flux (PWF) are summarized in Figure 4.

The obtained results confirm the positive effect of UV/O_3_ pretreatment on MF process performance. In case of the as-received MBBR effluent an increase of TMP above 1 bar did not result in any flux increase. On the opposite, for the UV/O_3_ pretreated wastewater the flux increased linearly with increasing TMP and was only ca. 30% lower than PWF. For comparison, the flux measured for the MBBR effluent at TMP = 3 bar was about 77% lower than PWF and about 65% lower than that obtained during MF of the UV/O_3_ pretreated wastewater. The differences between the fluxes increased with increasing TMP. Nonetheless, even at the lowest TMP applied (0.5 bar) the fluxes recorded during filtration of the wastewater were slightly lower compared to PWF. These results differ from those obtained during the recent studies on UF of the HMBBR effluent pretreated by UV/O_3_ [17]. In the previous research, the UF permeate fluxes measured at TMP = 1 bar were equal to PWF, which indicated that they did not exceed the critical flux, i.e., the flux below which the membrane fouling does not occur [18]. The differences between the results obtained in the present study and the previous research are justified considering that membrane fouling is a flux-dependent phenomenon, and the permeate fluxes were much lower in the case of the discussed UF process [18] compared to MF applied in the present research. Furthermore, since the MF membranes are characterized by larger pores than the UF ones, their proneness to fouling is also higher [27,36]. Although the MF membrane applied in the present research was fouled to some extent (Figure 4), even in the case of the applied UV/O_3_ pretreatment, it should be emphasized that the permeate flux measured at TMP = 3 bar was about 3 times higher compared to MF of the as-received MBBR effluent.

The main factor that contributed to the observed fouling alleviation is the conversion of large molecules of organic compounds present in laundry wastewater to smaller ones as a result of their oxidation under the action of in situ generated ozone and UV irradiation. Seo et al. [37] proved that the ozonation was an effective approach to degrade the organic compounds in the domestic laundry wastewater due to a conversion of the molecules larger than 10,000 g/mol to smaller than 500 g/mol by ozone injection. A positive effect of pre-ozonation on a membrane fouling mitigation was already reported in the literature by other researchers [24]. In general, it is widely confirmed that the application of the various AOPs can decompose and mineralize organic compounds responsible for the gel layer or the filtration cake formation, which results in enhancement of the permeate flux [18].

**Figure 5:**
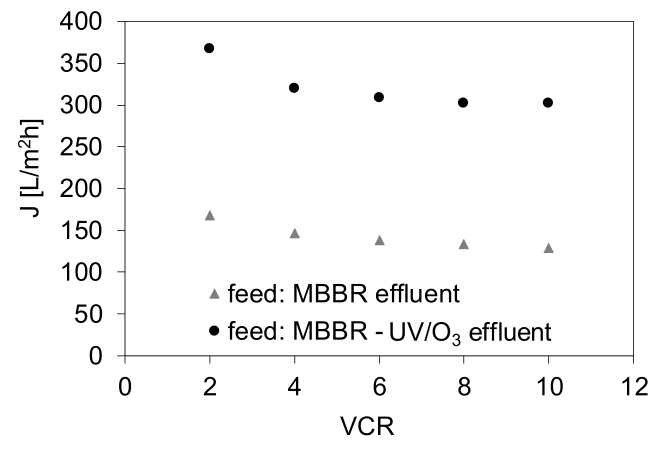
Influence of UV/O_3_ treatment on MF permeate flux for various VCR values (TMP = 1 bar in case of MBBR effluent and 2 bar in case of MBBR-UV/O_3_ effluent).

The positive effect of the applied UV/O_3_ pretreatment on the membrane fouling mitigation is even more noticeable when the permeate fluxes measured at various VCR values are compared (Figure 5). The experiment was realized in order to evaluate the possible water recovery in MF. In the case of MF of the as-received MBBR effluent the TMP of 1 bar was applied taking into account the results shown in Figure 4, revealing that further TMP increase did not result in any changes of a permeate flux. For MF of the MBBR-UV/O_3_ effluent the TMP = 2 bar was used to show the possibility of a significantly higher productivity under only small TMP increase. In case of both types of the feed it was possible to obtain a 90% recovery (VCR = 10). Nonetheless, the permeate fluxes were ca. 2.2–2.4 times higher when UV/O_3_ pretreatment was used compared to MF of the as-received MBBR effluent.

**Figure 6:**
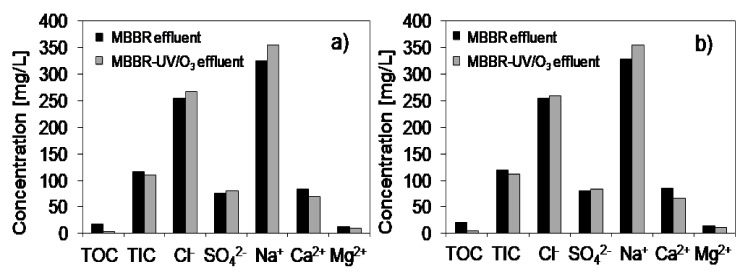
Influence of UV/O_3_ pretreatment on MF permeate quality at VCR = 2 (**a**) and 10 (**b**).

Based on the analysis of the composition of MF permeate collected at VCR = 2 and 10 for both systems (i.e., with and without UV/O_3_ oxidation) it was found that VCR had no significant effect on the quality of the product (Figure 6a,b). Moreover, in general, the loadings of the organic and inorganic substances in a permeate were similar in these two systems. The most significant difference refers to TOC concentration, associated with the mineralization of the organic contaminants upon the action of UV/O_3_. It can also be seen that a high amount of cations—such as Na^+^, Ca^2+^ and Mg^2+^—as well as anions (Cl^−^, SO_4_^2−^, and HCO_3_^−^ measured as TIC) remained in a permeate, what was reflected by its high conductivity (> 1800 μS/cm). Only turbidity in the case of both systems was reduced to the level acceptable for laundry water, i.e., 0.2–0.3 NTU. Since the water containing so high concentration of the inorganic ions could not be recycled to the laundry system, the nanofiltration was proposed as the final treatment step.

**Figure 7:**
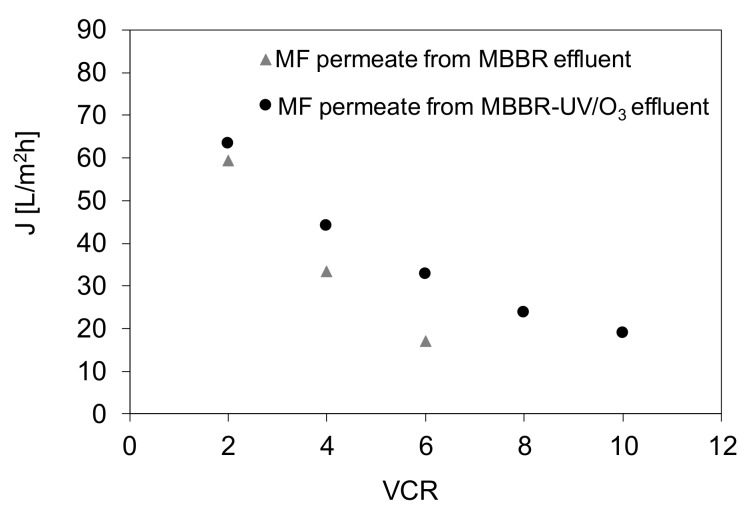
Influence of pretreatment procedure on NF permeate flux (TMP = 15 bar).

The application of the UV/O_3_ pretreatment had a positive impact on NF membrane fouling mitigation (Figure 7), even though in both systems (A and B) the MF process was applied before nanofiltration. The differences are especially noticeable at a water recovery rate of 75% or higher. At VCR = 4 the permeate flux was lower by ca. 25% and at VCR = 6 by ca. 50% when NF feed was pretreated by MBBR-MF system only. Due to the significant decrease of a permeate flux upon increasing the water recovery rate it was not possible to realize the NF at VCR higher than 6 when the effluent was not pretreated by UV/O_3_ process. On the opposite, the application of the photooxidation allowed to get a 90% water recovery, although the permeate flux under these conditions was by ca. 80% lower compared to the flux measured without any feed concentration.

The observed differences between the permeate fluxes shown in Figure 7 reveal that the decline of the water productivity was not only due to the increasing content of inorganic salts in a retentate upon increasing VCR, but also due to the presence of organic contaminants responsible for fouling. As was discussed before (Figure 3), the UV/O_3_ pretreatment allowed to decrease the TOC content by 68%, to the value of 6 mg/L. Thus, the concentration of organic foulants in the NF feed applied in system B was significantly lower compared to system A. Nonetheless, since both types of NF feed contained high concentrations of inorganic salts (initial conductivity > 1800 μS/cm) and these values increased with the increase of VCR, a deterioration of permeate flux was observed in both systems (Figure 7). That decrease was due to increasing osmotic pressure, which contributed to a decrease of the driving force [38].

The VCR value affected also the quality of the NF permeate (Table 3). In general, the concentration of the organic and inorganic species in a permeate increased with the increase of water recovery rates. This resulted from higher loadings of the contaminants in the more concentrated retentate. Moreover, in the case of monovalent ions (Na^+^, K^+^, Cl^−^) the Donnan effect influenced the separation efficiency. A negative impact of VCR on separation of ions, especially chlorides, in NF was also reported previously [39].

**Figure 8:**
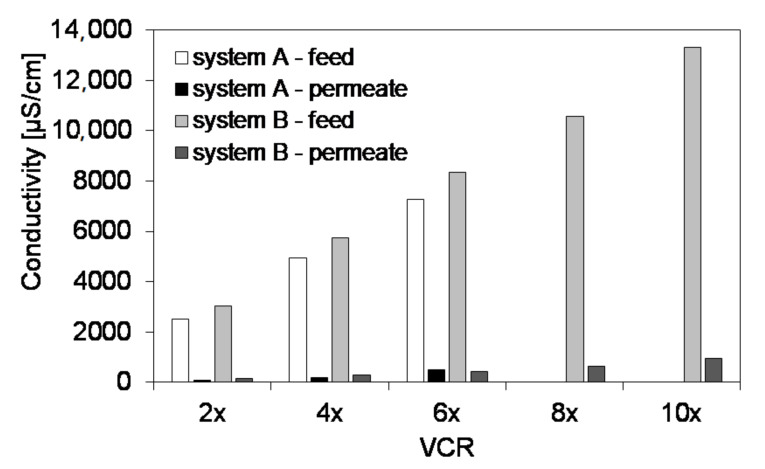
Comparison of conductivity of feed and permeate for various VCR in systems A and B.

Figure 8 presents, as an example, the changes of conductivity of feed and permeate upon increasing VCR in both systems. The rejection of the contaminants measured by means of this parameter was in the range of 93–97% and 93–95% for systems A and B, respectively. The lowest rejection was observed for the highest VCR values. Since the major ions in the NF feed were Cl^−^ and Na^+^ (Figure 6), the changes of conductivity can be mainly ascribed to these species.

Based on the composition of NF permeate collected at water recovery rates in the range of 50–83% in systems A and B in comparison with the quality of the water used in the industrial laundry (Table 2), it can be observed that application of UV/O_3_ as a pretreatment stage allowed to decrease the TOC content in the product. The values of the other parameters were comparable in both systems, although the NF feed in system A had slightly lower levels of contaminants measured as conductivity compared to that of system B (Figure 8) which was due to two different batches of raw laundry wastewater being used in the experiments. Comparing the quality of a permeate collected at VCR = 6 with that of the laundry water, it can be observed that only the concentration of Cl^−^ was noticeably higher in the NF product which was due to the presence of the wastewater stream from regeneration of ion exchangers. The change of the water softening system in the laundry into membrane one (e.g., NF) would solve this problem. Alternatively, mixing of NF permeate with the currently used laundry water would allow to decrease the Cl^−^ content if their elevated level becomes problematic (e.g., in the case of washing silk). It is also worth noting that the concentration of SO_4_^2−^ in the NF permeate was two orders of magnitude lower compared to that in the laundry water indicating the advantages of NF over conventional water treatment.

**Figure 9:**
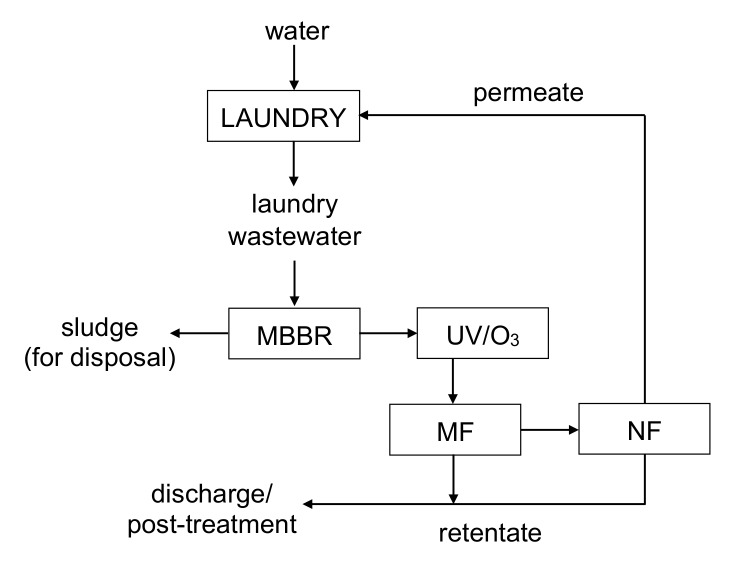
Schematic diagram of the proposed water cycle in laundry utilizing treatment and reuse of the laundry wastewater.

Since, in general, the water recovered from the wastewater treated by MBBR-UV/O_3_-MF-NF system exhibited quality comparable to the water used in the laundry, it was concluded that the NF permeate could be recycled to any stage of the laundry process (i.e., either washing or rinsing). A proposed scheme for water renovation and reuse in industrial laundry processes is shown in Figure 9.

It is worth noting that although a relatively high water recovery can be obtained in the proposed system (Figure 5 and Figure 7), some wastewater (MF/NF retentates) and sludge (from MBBR) will need utilization. An advantage of MBBR is reduced sludge production, thus the simplest solution is its disposal in the off-site sewage treatment plant. The MF and NF retentates can be mixed together and also disposed in a municipal wastewater treatment plant. One problem can be the presence of NaCl. The most beneficial solution for that could be to change the water softening technology from ion exchange to membrane technology. Alternatively, the recovery of NaCl from NF retentate and its reuse for ion exchanger regeneration can be considered.

The obtained results led to the conclusion that the proposed system (MBBR + UV/O_3_ + MF + NF) can be applied to recover water in the laundry. However, even the use of biological technology alone allowed to significantly decrease the content of surfactants, which concentration in the outflow of the MBBR was in the range of 0.4–3.6 mg/L. A similar value (< 2 mg/L) was obtained in a much more complicated system described in the subject literature [9], which included preliminary coagulation, followed by dissolved air flotation (DAF), sand filtration, ozonation, granulated activated carbon (GAC) filtration, and finally ultrafiltration. It was also proved that photolysis enhanced with in situ generated O_3_ (UV/O_3_) had a positive influence on MF and NF fouling mitigation. A similar effect was obtained [32] when UV/H_2_O_2_ treatment followed by MF using ceramic membrane was applied for the treatment of wastewater containing soluble algal organic matter. These data confirm a great potential of AOP in minimization of membrane fouling.

## 4. Conclusions

A three-stage hybrid system comprising biological (MBBR), AOP (UV/O_3_), and membrane (MF followed by NF) treatment steps was proposed for the treatment and reuse of industrial laundry wastewater. Although the removal efficiency in MBBR was high—reaching 95–97% for BOD_5_, 90–93% for COD, and 89–99% for anionic and nonionic surfactants—the post-treatment of the effluent was necessary before a possible reuse of the wastewater. The UV/O_3_ pretreatment prior to MF resulted in a membrane fouling mitigation in the case of both MF and NF processes. During MF, a linear increase of permeate flux with increasing TMP was observed when UV/O_3_ pretreatment was applied, while without pre-oxidation an increase of TMP above 1 bar did not result in any increase of the flux. Furthermore, in the whole range of the VCR values examined (2–10), the MF permeate flux was higher when UV/O_3_ pretreatment was used compared to the process without AOP pretreatment. MF was efficient in the removal of turbidity; however, to recycle the wastewater to the laundry process, further purification in terms of removal of TOC and inorganic contaminants—mainly Ca^2+^, Mg^2+^, Na^+^ and Cl^−^—was necessary. All of these contaminants were efficiently rejected by the NF membrane. The application of the photooxidation enabled higher water recovery in NF compared to the MBBR-MF pretreatment alone. The quality of the NF permeate was comparable to the quality of the water applied in the laundry, therefore, the obtained product could be recycled to any stage of the laundry process.

The obtained results suggest the efficacy of a general concept of a hybrid technology aimed at treatment and reuse of laundry wastewater. However, further investigations aimed at optimization of the unit operations and evaluation of the effect of changes of wastewater quality on product composition are necessary.

## Figures and Tables

**Table 1:** Composition of laundry wastewater before and after biological treatment.

Parameter	Unit	Before	After	Removal (%)
pH	pH	8.2	8.3–8.4	-
BOD_5_	mgO_2_/L	370–390	10–18	95–97
COD	mgO_2_/L	631–768	54–60	90–93
Total P	mgP/L	4.0–4.7	1.8–2.4	40–61
Total N	mgN/L	10–11	2.3–5.6	44–79
Surfactants				
anionic	mg/L	8.9–21.1	0.1–2.4	89–99
nonionic	mg/L	45.6–60.8	0.3–1.2	98–99

**Table 2:** Composition of laundry wastewater after biological treatment applied as a feedstock stream in systems A and B.

Parameter	Unit	Before System A (MF→NF)	Before System B (UV/O_3_→MF→NF)
TOC	mgC/L	19.4	18.6
TIC	mgC/L	103	127
Conductivity	μS/cm	1850	1969
Cl^−^	mg/L	264	261
SO_4_^2−^	mg/L	82	82
Na^+^	mg/L	326	344
K^+^	mg/L	14	17
Ca^2+^	mg/L	91.5	73
Mg^2+^	mg/L	12	9.3

**Table 3:** Influence of volume concentration ratio on NF permeate quality in system A (MBBR-MF pretreatment of NF feed) and B (MBBR-UV/O_3_-MF pretreatment of NF feed).

Parameter	Unit	VCR (−)/Water Recovery (%)						Laundry Water
		2x/50		4x/75		6x/83		
		A	B	A	B	A	B	
TOC	mgC/L	1.6	1.2	2.5	1.3	3.1	1.4	1.6
TIC	mgC/L	3.6	7.0	8.5	12	19	17	39
TDS	ppm	45	96	130	201	329	293	373
Cond.	μS/cm	70	150	200	305	489	436	550
Cl^−^	mg/L	12	27	38	58	96	87	27
SO_4_^2−^	mg/L	0.2	0.5	0.3	0.6	1	0.7	63
PO_4_^3−^	mg/L	<b.d.l.^#^	<b.d.l.	<b.d.l.	<b.d.l.	<b.d.l.	<b.d.l.	0.05
Na^+^	mg/L	14	30	40	62	100	88	131
K^+^	mg/L	1.1	1.1	1.8	2.2	4.6	3.4	2.6
Ca^2+^	mg/L	0.1	0.5	0.5	0.5	1.6	0.5	2.3
Mg^2+^	mg/L	<b.d.l.	0.1	0.1	0.1	0.3	0.2	0.1

**^#^** b.d.l. is short of below detection limit.
